# Exposure to a SARS-CoV-2 infection at work: development of an international job exposure matrix (COVID-19-JEM)

**DOI:** 10.5271/sjweh.3998

**Published:** 2021-12-30

**Authors:** Karen M Oude Hengel, Alex Burdorf, Anjoeka Pronk, Vivi Schlünssen, Zara A Stokholm, Henrik A Kolstad, Karin van Veldhoven, Ioannis Basinas, Martie van Tongeren, Susan Peters

**Affiliations:** 1 Netherlands Organisation for Applied Scientific Research TNO, Unit Healthy Living, Leiden, The Netherlands; 2 Erasmus Medical Center Rotterdam, Department of Public Health, Rotterdam, The Netherlands.; 3 Department of Public Health, Environment, Occupation and Health, Danish Ramazzini Centre, Aarhus University, Aarhus C, Denmark; 4 National Research Centre for the Working Environment, Copenhagen, Denmark; 5 Department of Occupational Medicine, Danish Ramazzini Centre, Aarhus University Hospital, Aarhus N, Denmark; 6 London School of Hygiene & Tropical Medicine, Faculty of Epidemiology and Public Health, London, UK; 7 Centre for Occupational and Environmental Health; Centre for Epidemiology; Division of Population Health, Health Services Research and Primary Care; School of Health Sciences; Faculty of Biology, Medicine and Health, The University of Manchester, Manchester Academic Health Science Centre, Manchester, UK; 8 Environmental Epidemiology Division, Institute for Risk Assessment Sciences, Utrecht University, Utrecht, The Netherlands

**Keywords:** COVID-19, mitigation factor, precarious work, transmission risk, variance

## Abstract

**Objective:**

This study aimed to construct a job exposure matrix (JEM) for risk of becoming infected with the SARS-CoV-2 virus in an occupational setting.

**Methods:**

Experts in occupational epidemiology from three European countries (Denmark, The Netherlands and the United Kingdom) defined the relevant exposure and workplace characteristics with regard to possible exposure to the SARS-CoV-2 virus. In an iterative process, experts rated the different dimensions of the COVID-19-JEM for each job title within the International Standard Classification of Occupations system 2008 (ISCO-08). Agreement scores, weighted kappas, and variances were estimated.

**Results:**

The COVID-19-JEM contains four determinants of transmission risk [number of people, nature of contacts, contaminated workspaces and location (indoors or outdoors)], two mitigation measures (social distancing and face covering), and two factors for precarious work (income insecurity and proportion of migrants). Agreement scores ranged from 0.27 [95% confidence interval (CI) 0.25–0.29] for ‘migrants’ to 0.76 (95% CI 0.74–0.78) for ‘nature of contacts’. Weighted kappas indicated moderate-to-good agreement for all dimensions [ranging from 0.60 (95% CI 0.60–0.60) for ‘face covering’ to 0.80 (95% CI 0.80–0.80) for ‘contaminated workspaces’], except for ‘migrants’ (0.14 (95% CI -0.07–0.36). As country differences remained after several consensus exercises, the COVID-19-JEM also has a country-axis.

**Conclusions:**

The COVID-19-JEM assesses the risk at population level using eight dimensions related to SARS-COV-2 infections at work and will improve our ability to investigate work-related risk factors in epidemiological studies. The dimensions of the COVID-19-JEM could also be valuable for other future communicable diseases in the workplace.

After a new type of coronavirus (SARS-CoV-2) surfaced at the end of 2019, the incidence of coronavirus-disease-2019 (COVID-19) rapidly increased with a substantial number of fatalities across all countries in the world (1). Governments implemented measures to change population behaviors and limit social contacts outside the household to curb the infection curve. The so-called ‘lockdowns’ have far-reaching consequences, also for the working population. Worldwide, workers were encouraged to work from home whenever possible, while essential workers – who are vital for the core function of the society – remained at work, and therefore potentially in contact with co-workers, members of the general public or patients, and thus potentially exposed to the virus. Closure of specific sectors (eg, education, entertainment industry, and accommodation and food service activities) for certain periods was also implemented, and the closure patterns differed between and within countries over time (2). In between the periods of lockdowns, some occupations such as teachers, hairdressers, waiters and retailers went back to work, while other occupations were encouraged to continue to work from home.

For workers in occupations where it is not able to work from home, the workplace will contribute to the overall risk of becoming infected with SARS-CoV-2 (3). A study in the United States showed that approximately 10% of all workers are employed in occupations where exposure to disease or infection occurs at least once per week (4). The risk of getting infected with SARS-CoV-2 depends on the potential of being in contact with infected people, the characteristics of the work environment (eg, inside/outside, ventilation) and the presence of mitigating measures such as distancing and personal protective equipment. For example, a rapid increase in SARS-CoV-2 infections during the first months of the pandemic was observed amongst frontline healthcare workers (5–9). However, due to the increasing availability and prevailing use of face coverings and other preventive measures, widespread nosocomial transmission between workers and patients reduced (10, 11). Besides healthcare workers, there is a long list of jobs in which workers are at increased risk of a SARS-CoV-2 infection, usually because their activities require close proximity to the general public. For example, security guards and taxi drivers had the highest mortality rates of all workers during the first weeks of the COVID-19 pandemic in England (12). Jobs reported to have high a risk of infection included hairdressers and public transport drivers in The Netherlands (13) and bartenders, transport conductors and travel stewards in Norway (11).

In addition, COVID-19 outbreaks were described in essential sectors, such as agriculture and meat processing, where many (migrant) workers face poor working conditions. This may often involve working in close proximity with each other with no or limited protective measures, with limited test capacity and working and where workers may also share travel and domestic arrangements (14–17). Workers with such precarious jobs (18) may be at higher risk of being infected with SARS-CoV-2 due to financial barriers that may reduce ability to self-isolate.

Workplaces may be one of the key settings in the spread of SARS-COV-2 infections, among both essential and non-essential workers. It is therefore important to assess the occupations at increased risk of exposure to SARS-CoV-2 in large study populations. A job exposure matrix (JEM) is a common tool to classify job titles by degree of occupational exposure to a potential health hazard in epidemiological studies (19, 20). As obtaining exposure data on SARS-CoV-2 at the individual level is difficult in many countries, if not impossible considering the time scales involved, a specific JEM for the occupational exposure to SARS-CoV-2 can be useful as a quick and systematic means of converting occupations into estimates of exposure. Such a JEM will enhance the investigation of the role of the workplace in the spread of the SARS-CoV-2 infection and subsequent cases of COVID-19 disease. Moreover, insight into occupations at higher risk of becoming infected with an airborne virus due to the working conditions (ie, risk for transmission and mitigation measures) can also be valuable in relation to influenza or other potential airborne spread diseases (21). In addition to national attempts to estimate the risk of SARS-CoV-2 infection in specific occupations (4, 7, 11, 13) or development of national JEM (22), there is a need to construct a harmonized JEM that is applicable across various countries. Therefore, the aim of the current study was to describe the development of an international JEM for jobs with an increasing risk of cases of COVID-19, the COVID-19-JEM.

## Methods

### Expert group

A JEM for the risk of becoming infected with the SARS-CoV-2 virus was constructed based on expert assessment and national data. Ten experts in occupational epidemiology and exposure assessment from three different European countries (ie, The Netherlands, Denmark and the United Kingdom) were involved. Three members of the expert team drafted the initial proposal for relevant exposure and workplace characteristics to be included in the COVID-19-JEM. All members of the expert group were involved in the subsequent consensus discussions towards finalizing this proposal to establish the relevant COVID-19-JEM dimensions and their interpretation, as well as the corresponding risk ratings required. The risk ratings, explained in more detail below, were independently provided by nine of the experts. Regular online meetings were organized to guarantee efficient communication and consensus agreements within and between countries.

### Framework for constructing the COVID-19-JEM

The framework for developing the COVID-19-JEM was based on four principles.

*Number, nature of contacts and proximity of contact*. Workers face higher risks of becoming infected when working in close proximity to each other (eg, construction worker, meatpacker), and/or members of the public (eg, hairdresser, teacher), and/or patients with (suspicion of) COVID-19 (eg, healthcare worker). Thus, the COVID-19-JEM should take into account the nature and frequency of daily contacts with other persons (23).

*Work location*. Transmission patterns may be influenced by the working environment. It is obvious that working from home will reduce transmission. Likewise, working outdoors may reduce the risk of transmission compared to working indoors, especially when ventilation is poor.

*Mitigation measures*. The risk for COVID-19 depends also on the prevention and mitigation measures available and implemented. Control measures of interest are social distancing and the use of face covering.

*Precarious work*. The work environment will be influenced by the employment relationship. In the context of the COVID-19-JEM, precarious work is of particular interest as temporary jobs, multiple jobs, and/or insecure jobs typically involve poorer working conditions (24) and an increased risk for less stringent enforcement of mitigation actions. Similarly, migrants are often employed in precarious work, and their risk of becoming infected may be amplified by poor housing and commuting conditions, such as crowding and inadequate ventilation (25).

### Dimensions in the COVID-19-JEM

The above principles were translated into eight dimensions within the COVID-19-JEM: four determinants of transmission risk, two mitigation measures, and two factors on precarious work.

The first determinant of transmission risk captured the number of fellow workers in close vicinity to each other on a regular workday. The second dimension focused on the nature of contacts, which can be co-workers, the general public or patients with (suspected) COVID-19, while the third dimension addressed the frequency and nature of contact with potentially contaminated work surfaces and materials. The fourth determinant of transmission was the working environment – ie, whether working in- or outdoors for part or most of the workday.

The mitigation measures distinguished social distancing and use of face coverings. Social distancing was defined as maintaining a distancing of ≥1 meter between colleagues or members of the public while at work, as advised by the WHO (26). The face covering dimension assessed the likelihood that workers wear face coverings whilst working in close proximity to colleagues or members of the public. Face coverings were considered to prevent or reduce the spread of infection and could include surgical masks, face shields or similar equipment. Some workers will also have access to respiratory protective equipment (RPE), and the assessment determines the likelihood that face covering or RPE is used during interaction with co-workers, general public or patients.

Precarious work is a multifaceted concept, and we focused on income insecurity and first-generation migrants as key aspects of precarious employment (18). For each job title, the income insecurity was rated as proportion of workers with a flexible labor contract, defined as a type of contract where a national or local mitigation measure, such as lockdown of bars, restaurants, and shops, would result in a drop in disposable personal income at the short-term. This may include zero-hour contracts, casual work, and day labor. The second dimension is the proportion of migrants in each job, whereby we did not distinguish by educational level.

For each of the four determinants of transmission risk and two mitigation measures, the level of risk was rated at four levels: no, low, elevated, and high risk. Specific rules were developed to guide the expert rater in classifying the risk (table 1). Both factors of precarious workers were also categorized into four levels, based on the proportion per job title: <1, 1–10, 11–25 or ≥25%. With regard to precarious work, experts from the UK relied on data from national statistics to estimate the risks per job title, whereas the estimates in Denmark and The Netherlands relied on expert’s assessment as objective data on precarious work could not be easily distracted in these countries.

**Table 1:** The dimensions, descriptions and risk categories of the COVID-19-JEM: four determinants of transmission risk, two mitigation measures, and two factors related to precarious work.

COVID-19-JEM		Risk score			
	
Dimension	Description	No risk (score=0)	Low risk (score=1)	Elevated risk (score=2)	High risk (score=3)
Transmission risk					
Number	The number of workers in close vicinity of each other	Homeworkers, or not working with others	<10 per day	10–30 per day	>30 per day
Nature of contacts	The nature of contacts with co-workers, general public or patients with COVID-19	Homeworkers, or not working with others	Working in workspaces with co-workers only	Working in workspaces with general public	Working in workspaces with regular contacts with suspected or diagnosed COVID-19 patients
Contaminated workspaces	The risk through contaminated work surfaces and materials	Homeworkers, or not working with others	Frequently sharing materials/surfaces with co-workers (≥10 times a day)	Sometimes sharing materials/surfaces with general public (<10 times a day)	Frequently sharing materials/surfaces with general public (≥10 times a day)
Location	Indoors or outdoors	Homeworkers, or not working with others	Mostly working outside	Working partly inside (1–4 hours/day)	Working mostly inside (>4 hours/day)
Mitigation factors					
Social distancing	The possibility to keep ≥1m of social distance	Homeworkers, or not working with others	Social distancing can always be maintained	Social distancing cannot always be maintained	Social distancing can never be maintained
Face covering	The need and usage face covering	Homeworkers, or not working with others	Wearing face covering at the worksite	Wearing face covering during specific activities, but not always while in proximity of others	Activities in proximity of others which cannot be done when wearing face covering (eg, sports, singing)
Precarious work					
Income insecurity	Proportion of income insecurity due to the pandemic	<1%	1–10%	11–25%	>25%
Migrants	Proportion of labor migrants	<1%	1–10%	11–25%	>25%

Cut-off values for the risks per dimension and rules were developed based on consensus among all experts.

### Default setting

As the COVID-19 pandemic and governmental measures differ over time and between countries, a default setting was defined relevant to the situation to which the COVID-19-JEM refers. This default setting was defined as the situation where general mitigation measures are present (social distancing, washing hands, face covering) but the country is not under full lockdown and where vaccination has not started yet. In other words, hairdresser, construction workers and teachers are working at the worksite, while most office workers are still required to work from home.

### Expert assessment step-by-step

The COVID-19-JEM was developed based on the International Standard Classification of Occupations 2008 (ISCO-08) coding scheme with four-digit codes describing 436 job titles. The iterative development process consisted of four steps and a standardized protocol was developed to follow throughout the implementation of every step.

### Independent expert rating (step 1)

The experts independently rated all six dimensions of the risk of transmission and mitigation measures of the COVID-19-JEM for each job title included in ISCO-08. As the risk to be infected with SARS-CoV-2 might differ among workers within sub-industries of especially the healthcare sector, the sublevel of industry according to the nomenclature of economic activities (NACE) classification (8610–8890) (27) was added to seven jobs titles (2221, 2240, 2269, 2635, 3253, 3256, 3259).

### Group expert meeting and revision (step 2)

All experts discussed the difficulties in the interpretation of the dimensions for the risk of transmission and mitigation measures overall and within specific job titles. If too many uncertainties were present, the definition of the dimensions involved were reconsidered and tailored accordingly. Part of this process was the provision of concrete examples regarding job titles where uncertainties were experienced. Afterwards, all experts independently revised their individual ratings where needed.

### Independent country rating (step 3)

Once the revised individual ratings were obtained, meetings for discussing differences and reaching consensus between the experts within each country were established. Rules on consensus per job title and dimension were established a priori. For differences in risk scores of one point between any pair of raters, and if all country raters deemed the job to be exposed, the majority rating was applied. If there was disagreement whether the job should be classified as unexposed, a discussion to reach consensus followed. For differences of ≥2 points, the majority rule was applied as default, but the score could be adapted based on the discussions among the raters.

### Consensus between countries (step 4)

Lastly, a meeting involving one expert per country was held to discuss the observed differences between countries, primarily concerning assessments of job titles perceived as non-exposed in some countries and as exposed in others. Discussions focused on whether the observed differences were reflecting actual differences between the countries or whether they were the result of different interpretations. In the latter case, the assessment was reconsidered within each country.

Additionally, three experts discussed the definitions for the two factors on precarious work after comparing preliminary results from each country. Thereafter, each country independently provided revised input for the two factors on precarious work.

### Statistical analyses

For each step, the mean, standard deviation (SD) and variance components of the assigned ratings per job title were calculated. Additionally, three performance indicators were used to evaluate the reliability and agreement between raters: (i) an agreement score, (ii) the weighted kappa, and (iii) the variance. These indicators were estimated as overall score between and within each country for each dimension (ie, raters nested within countries). An agreement score can range from 0 (0%) to 1 (100%) where the latter means total agreement between experts (28). The weighted kappa coefficient also measures agreement but takes into account that agreement may occur by chance. Kappa values were classified according to Cohen, as follows: poor (<0.20), fair (0.21–0.40), moderate (0.41–0.60), good (0.61–0.80) and excellent (0.81–1) agreement (29).

For step 1 (ie, independent expert rating) and step 2 (group expert meeting and revision), agreements scores and kappas were estimated overall and per country. A hierarchical analysis of variance was conducted to determine which level contributes most to the observed variance. The rankings of three raters per country were nested within job titles to provide insight into within-job variance due to raters and between-job variance for each country. Subsequently, job titles were also nested within country to evaluate whether job rankings differed across countries. This approach allowed components of variance to be attributed to raters, jobs, and countries.

Because step 3 (independent country rating) and step 4 (consensus among countries) included one risk score for each country, agreement scores and weighted kappas were only estimated overall as the variance could only distinguish variance by job title and country. In step 4, this procedure was also conducted for the two factors of precarious work.

All estimations were conducted in R version 4.0.2.

## Results

**Figure 1:**
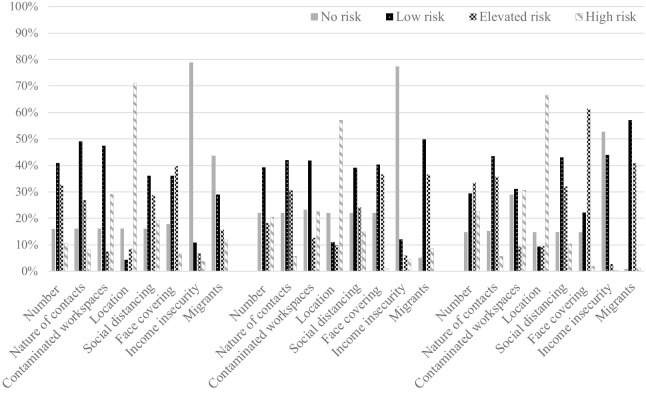
The proportion of jobs per risk category for all eight dimensions in each country.

The definitions and risk scores assignments of the COVID-19-JEM including the eight dimensions are presented in table 1. The final risk scores on the eight dimensions of the COVID-19-JEM are presented for all three countries separately (supplementary material, https://www.sjweh.fi/article/3998, tables S1.1–S1.3). Figure 1 shows the proportion of jobs in each risk category for all eight dimensions. The proportion of job titles with a high risk was the largest for ‘location’ and ‘contaminated workspaces’ across all countries. The proportion of job titles rated as non-exposed based on transmission and mitigation factors were the largest in The Netherlands, except for contaminated workspaces which was the largest in the UK. Denmark showed the smallest proportions of precarious work in the job titles. As an illustration of the ratings, table 2 shows six job titles and their risk for each dimension in each country.

**Table 2:** Examples of six job titles and their risk within each of the eight dimensions of the COVID-19-JEM (all job titles are presented in supplementary file 3-5). [DK=Denmark; NL=The Netherlands; UK=United Kingdom]

ISCO-08	Job title	Country	COVID-19 JEM							

			Transmission risk ^a^				Mitigation factors ^a^		Precarious work ^b^	
		
			Number	Nature of contacts	Contaminated workspaces	Location	Social distancing	Face covering	Income insecurity	Migrants
1211	Finance managers	DK	0	0	0	0	0	0	0	0
1211	Finance managers	NL	0	0	0	0	0	0	0	2
1211	Finance managers	UK	0	0	0	0	0	0	0	1
2221	Nursing professionals	DK	3	3	3	3	3	1	0	0
2221	Nursing professionals	NL	3	3	3	3	3	1	0	1
2221	Nursing professionals	UK	3	3	3	3	3	1	1	1
4214	Debt-collectors and related workers	DK	0	0	0	0	0	0	0	1
4214	Debt-collectors and related workers	NL	1	2	2	3	1	1	0	1
4214	Debt-collectors and related workers	UK	2	2	2	3	1	1	0	2
5152	Domestic housekeepers	DK	1	2	3	3	2	2	2	3
5152	Domestic housekeepers	NL	1	2	3	3	2	2	2	2
5152	Domestic housekeepers	UK	1	2	2	3	2	2	0	2
5221	Shop keepers	DK	3	2	3	3	3	1	2	2
5221	Shop keepers	NL	3	2	3	3	2	1	2	1
5221	Shop keepers	UK	3	2	3	3	2	1	0	1
7112	Bricklayers and related workers	DK	1	1	1	2	2	2	0	3
7112	Bricklayers and related workers	NL	2	1	1	1	2	2	0	1
7112	Bricklayers and related workers	UK	1	1	1	2	2	1	1	2
8322	Car, taxi and van drivers	DK	3	2	3	3	3	2	2	3
8322	Car, taxi and van drivers	NL	3	2	2	3	2	1	2	1
8322	Car, taxi and van drivers	UK	3	2	3	3	2	2	1	2

a Transmission risk and mitigation factors are assessed with a risk score (0-3) to occupational exposure (see table 1 for details).

b Precarious work is assessed with a score of 0–3 (0=<1%, 1=1–10%, 2=11–25%, 3=>25%).

### Independent expert rating (step 1)

The agreement scores of the independent expert ratings ranged from 0.46 (95% CI 0.46–0.49) for ‘face covering’ to 0.71 (95% CI 0.69–0.74) for ‘location’ (table 3). The lower agreement in ‘face covering’ is also reflected by the variance, as 67.0% of the variance was attributed to differences between raters and 14.0% to differences between countries. The performance of the COVID-19-JEM for the other five dimensions (number of people, nature of contacts, contaminated workspaces, location and social distancing) was moderate with ≥50% of the variance at job level. Weighted kappas varied between poor [0.17 (95% CI 0.12–0.22) for ‘face covering’)] to good [0.67 (95% CI 0.67-0.67 for ‘social distancing’)].

**Table 3:** Agreement scores, weighted kappas, total and variance for each step of the development process. [CI=confidence intervals].

	Agreement score (95% CI)	Weighted kappa (95% CI)	Total variance	Variance of total variance (%)		

				Country	Job	Rater
Independent expert rating (Step 1)						
Number	0.49 (0.46–0.51)	0.51 (0.49–0.52)	1.02	0.0	51.1	48.9
Nature of contacts	0.70 (0.67–0.72)	0.64 (0.64–0.64)	0.69	0.3	64.3	35.3
Contaminated workspaces	0.59 (0.57–0.62)	0.65 (0.64–0.66)	1.21	0.0	65.1	34.9
Location	0.71 (0.69–0.74)	0.56 (0.55–0.58)	1.50	2.3	55.9	41.8
Social distancing	0.58 (0.55–0.60)	0.67 (0.67–0.67)	0.88	0.0	67.1	32.9
Face covering	0.46 (0.44–0.49)	0.17 (0.12–0.22)	0.76	14.0	19.0	67.0
Group expert meeting and revision (Step 2)						
Number	0.50 (0.48–0.53)	0.54 (0.52–0.55)	1.05	0.0	54.1	45.9
Nature of contacts	0.68 (0.66–0.70)	0.63 (0.63–0.63)	0.73	0.0	63.5	36.6
Contaminated workspaces	0.59 (0.57–0.62)	0.63 (0.62–0.64)	1.20	0.0	62.8	37.1
Location	0.71 (0.68–0.73)	0.55 (0.54–0.56)	1.54	0.7	55.0	44.4
Social distancing	0.54 (0.52–0.57)	0.66 (0.65–0.66)	0.94	0.2	65.6	34.1
Face covering	0.54 (0.51–0.56)	0.34 (0.31–0.37)	0.79	7.5	34.3	58.2
Independent country rating (Step 3)						
Number	0.55 (0.53–0.58)	0.60 (0.56–0.64)	0.99	2.7	97.3	
Nature of contacts	0.74 (0.71–0.76)	0.70 (0.70–0.70)	0.68	0.0	100.0	
Contaminated workspaces	0.68 (0.66–0.71)	0.75 (0.75–0.75)	1.27	0.0	100.0	
Location	0.74 (0.72–0.76)	0.55 (0.50–0.60)	1.43	0.9	99.2	
Social distancing	0.63 (0.60–0.65)	0.74 (0.74–0.74)	0.89	0.0	100.0	
Face covering	0.70 (0.67–0.72)	0.56 (0.50–0.61)	0.67	2.0	97.9	
Consensus between countries (Step 4)						
Number	0.58 (0.55–0.61)	0.65 (0.63–0.68)	0.97	0.3	99.5	
Nature of contacts	0.76 (0.74–0.78)	0.76 (0.76–0.76)	0.68	0.4	99.6	
Contaminated workspaces	0.70 (0.68–0.73)	0.80 (0.80–0.80)	1.25	1.9	98.0	
Location	0.77 (0.75–0.79)	0.65 (0.64–0.66)	1.40	0.8	98.9	
Social distancing	0.66 (0.63–0.68)	0.79 (0.79–0.79)	0.89	0.0	95.7	
Face covering	0.72 (0.69–0.74)	0.60 (0.60–0.60)	0.66	0.3	99.5	
Income insecurity	0.66 (0.64–0.69)	0.53 (0.42–0.64)	0.52	1.2	98.8	
Migrants	0.27 (0.25–0.29)	0.14 (-0.07–0.36)	0.71	4.8	95.2	

Patterns for agreement scores were similar in all countries (supplementary table S2.2). The highest agreement scores were found for ‘nature of contacts’ and ‘location’ in all countries and the lowest for ‘face covering’ in Denmark [0.40 (95% CI 0.37–0.42)] and The Netherlands [0.39 (95% CI 0.37–0.42)]. The lowest variance by job group level was for ‘face covering’ in Denmark (9.2%) and The Netherlands (23.9%).

### Group expert meeting and revision (step 2)

During the group meeting a need for adjustment of the definitions for the ‘face covering’ and ‘number of people’ dimensions was recognized and applied. This revision resulted in generally in slightly changes on the agreement scores, weighted kappas and variance components overall (table 3).

The agreement score for ‘face covering’ increased to moderate [0.54 (95% CI 0.51–0.56)] and the kappa increased to fair [0.34 (95% CI 0.31–0.37)]. The variance by job title was 34%, mainly due to higher variance at job level in Denmark and The Netherlands (supplementary table S2.3).

### Independent country rating (step 3)

After the consensus meeting within each country, agreements scores improved for all four dimensions of risk transmission and two mitigation measures, with the largest improvement for ‘face covering’ (0.70 (95% CI 0.67–0.72); table 3). Weighted kappas ranged from moderate [0.55 (95% CI 0.50–0.60 for ‘location’)] to good [0.75 (95% CI 0.75–0.75) for ‘contaminated workspaces’]. The variance by job title reached 100% for nature of contacts, contaminated workspaces and social distancing.

### Consensus between countries (step 4)

Comparisons and discussions of differences in assessments between countries led to changes in scores for some job titles, whereas scores for the vast majority remained unchanged, reflecting some perceived actual differences in working conditions between countries. As an example, a debt collector was considered a home worker in Denmark, but not in The Netherlands or the UK (table 2).

This last step slightly improved the agreement scores and weighted kappas (table 3). ‘Number of people’ had the lowest agreement scores [0.58 (95% CI 0.55–0.61)] while ‘location’ had the highest agreement score [0.77 (95% CI 0.75–0.79)]. The weighted kappas ranged from moderate [0.60 (95% CI 0.60–0.60 for ‘face covering’)] to excellent [0.80 (95% CI 0.80–0.80 for ‘contaminated workspaces’)]. The variance by job group ranged from 96% for ‘social distancing’ tot 99.6% for ‘nature of contacts’.

In this step, the scores of precarious work were also added to the COVID-19-JEM. Due to the differences between countries, the agreement score [0.27 (95% CI 0.25–0.29)] and weighted kappa [0.14 (95% CI -0.07–0.36)] were poor for the dimension ‘migrants’, but both performance indicators were moderate for ‘income insecurity’ [agreement score of 0.66 (95% CI 0.64-0.69) and weighted kappa of 0.53 (95% CI 0.42–0.64); table 3].

## Discussion

The COVID-19-JEM contains four determinants of risk transmission (number of people, nature of contact, contaminated workspaces and location), two mitigation factors (social distancing and face covering) and two factors for precarious work (income insecurity and migrants). Based on an iterative process with four steps involving ten experts from three countries (Denmark, The Netherlands and the UK), all 436 job titles of the four-digit ISCO-08 coding scheme were assigned a risk score ranging from 0–3 for the eight dimensions. The final COVID-19-JEM generally showed moderate to good agreement between raters, except for the dimension ‘migrants’. Inter-rater reliability was good to excellent for most dimensions, except for ‘migrants’ and ‘face covering’ which showed a poor and moderate reliability, respectively.

As the occupational setting plays an important role in outbreaks of COVID-19 at a local level (30), constructing an accessible COVID-19-JEM is a first step to assess occupational risk at a population level. Within the process, improvements in agreement and inter-rated reliability appeared for each step of the development process, after in-depth discussions within and between countries. In general, the experts between countries agreed whether workers were exposed or not, but more often disagreed on the extent to which this exposure occurred. As it was acknowledged that some actual differences between countries exist, we did not attempt to develop one general COVID-19-JEM. Instead, the country specific assessments for Denmark, The Netherlands and the UK are presented separately. When applying the COVID-19-JEM in future research, researchers need to be aware of the differences between the country of interest and the countries included in this COVID-19-JEM. While transmission risk and mitigation measures are rather similar across countries, larger differences may be present in precarious work. For implementation, it is therefore recommended to select the country-axis with the highest similarities of the population under study on transmission risk and mitigation measures and to carefully investigate whether risk scores should be adapted for the country under study. With regard to precarious work, the scores need to be translated towards the specific country, or even specific province or state.

The final two dimensions of the COVID-19-JEM relate to precarious work were the proportion of workers with income insecurity due to the pandemic and the proportion of migrants. The importance of these factors was emphasized in a recent Canadian study, showing that workers in low-income occupations were at the highest risk of developing COVID-19 (31). It should be noted that the proportion of workers with income insecurity and the proportion of migrants might largely differ between countries and regions due to labor market regulation, economic composition and welfare systems (32). This is also reflected in the current study by the poor agreement and low weighted kappa for migrants between countries. Additionally, the UK used national statistics to provide input on the dimensions for precarious work and experts in The Netherlands and Denmark rated these dimensions by themselves, which might also influenced the differences between countries. Because of the large differences between country – and even regions within countries – it is recommended to adjust the precarious work factors of the selected COVID-19-JEM to the population under study. Furthermore, these findings suggest that the need for tailoring precarious work-related dimensions to the population/country at hand expands likely to multinational JEM for exposures other than COVID-19.

The current study described the development and presentation of the COVID-19-JEM, and the next step to take is the validation of the risk scores per job title and estimate the associations of these dimensions with the prevalence of infections per job title from large (administrative) observational data. A first small step on validation has been conducted in the UK, whereby the COVID-19-JEM risk scores were translated from the ISCO-08 to SOC2010 codes (33). These risk scores within each dimension were validated by comparing them with infection survey data from the Office of National Statistics (ONS). The preliminary results on this small dataset showed that a small increase in the proportion of COVID-19 infected persons was observed with increasing risk scores in each dimension of the COVID-19-JEM. When reliable objective data on sufficient numbers of COVID-19 infections from all three countries is available, research on the validation of the COVID-19-JEM will continue. To further encourage other researchers to apply the COVID-19-JEM, it is freely accessible through the OMEGA-NET website and presented in the supplementary material. Furthermore, researchers need to be aware that the COVID-19-JEM in the current study is developed assuming a “basic state” of the pandemic, in which general measures are taken (social distancing, washing hands, face covering in public places, working from home as possible) but without closure of sectors. As the COVID-19-JEM might need to be specified to accommodate local conditions and measures taken, table 4 describes an example on how a multiplier can be used in order to consider the severity of the pandemic. Additionally, the COVID-19-JEM is based on ISCO-08, which has often been used to classify jobs in large population-based studies. As the COVID-19-JEM may be applied in other studies using other occupational coding schemes such as previous versions of the ISCO, crosswalks can be used to link the ISCO-08 to other coding schemes.

**Table 4:** Example of rules to change the COVID-19-JEM depending on the state of the governmental measures.

No lockdown
Office workers work from home as much as possible (eg, secretaries, managers, ICT personnel) All workers who need to work at the workplace are allowed to work (eg, teachers, hairdressers, waiters, construction workers). Social distancing, washing hands and using face covering are required	Standard COVID-19-JEM as developed Standard COVID-19-JEM as developed Standard COVID-19-JEM as developed
Partial lockdown
Some industries are required to work less (eg, bars and restaurants need to close earlier) or needs to close entirely for a specific time period.	Closing earlier does not change the JEM Closing an industry has consequences for the JEM, all jobs related to this industry needs to be set to a risk of 0.
Total lockdown
Only essential workers can continue their work, while others are required to work from home or not to work.	Essential workers keep their JEM dimensions The risk for all other jobs will be 0

The major strength of this study is the collaboration between multiple experts from three European countries within an iterative process to develop a COVID-19-JEM, which resulted in improvement of agreement and reliability through the different steps. A JEM allows for a systematic translation of jobs into exposures, which makes a JEM a highly efficient and reproducible method (20). The same approach can be used in different study populations, and the assessment is fully documented so it is transparent and adaptations can be made easily when needed for a particular country, for example.

The current study, however, is not without limitations. Firstly, the expert judgement approach to construct a JEM has been criticized for lower validity as compared to direct measurement of exposure. However, as exposure data is not available on a larger scale yet, an expert judgement approach is the only option. To optimize validity, we constructed the COVID-19-JEM with an international team consisting of nine experts and by taking a structured four-step approach. Secondly, the COVID-19-JEM consists of four risk factors for transmission, two mitigations factors and two factors for precarious work. Each dimension of precarious work was defined as the proportion of workers within each job title. Even though survey or register data potentially have a higher validity, such data was only available in the United Kingdom. This data could not easily be distracted in The Netherlands and Denmark and an expert assessment was needed. Thirdly, even though some dimensions are probably more strongly related to an infection risk than others, and collinearity exists between dimensions, future validation research is needed in studies with large samples of the workforce in order to disentangle the particular contribution of the dimensions to SARS-COV-2 infection rates. Fourthly, several variants of the virus are already spreading across the world which might have a different impact on the spread of the pandemic (34). However, the assessment of the eight dimensions are generic and independent of the “potency” of SARS-COV-2. Finally, by design, a JEM assigns the same exposure to everyone with the same job title. Heterogeneity between workers, however, will be high for the dimensions that we assessed, particularly when it is largely dependent on workers’ behavior.

To conclude, the COVID-19-JEM is a first step in identifying occupations where workers are at risk of being infected with SARS-CoV-2. It consists of factors for transmission risk and mitigation as well as precarious work. The current study showed moderate-to-good agreement and inter-rater reliability on the different dimensions of the COVID-19-JEM. The COVID-19-JEM is a valuable tool for future epidemiological studies on SARS-COV-2 when individual data on relevant work conditions are lacking.

### Funding

KOH received funding from a VENI grant from The Netherlands Organisation for Scientific Research (NWO), project number 451-16-031.

The development of this JEM was conducted within the scope of OMEGA-NET, supported by COST (European Cooperation in Science and Technology), project CA16216: Network on the Coordination and Harmonisation of European Occupational Cohorts.

This study is partly funded by EPHOR (the European Union’s Horizon 2020 research and innovation programme under grant agreement No 874703).

The UK contribution to this work was supported by funding from the PROTECT COVID-19 National Core Study on transmission and environment, managed by the UK Health and Safety Executive on behalf of HM Government.

## Supplementary material

Risk scores on the eight dimensions of the COVID-19-JEM

Supplementary tables
