# Supplementary material for: Exposure to a SARS-CoV-2 infection at work: development of an international job exposure matrix (COVID-19-JEM)
Source: Scand J Work Environ Health. 2021 Dec 30;48(1):61–70. doi: 10.5271/sjweh.3998 (PMC8729167; doi:10.5271/sjweh.3998)
Supplement: Supplementary tables [file SJWEH-48-61-S002.pdf]

Exposure to a SARS-CoV-2 infection at work: development of an international job exposure matrix (COVID-19-JEM) [1]

by Karen M Oude Hengel, PhD, [2] Alex Burdorf, PhD, Anjoeka Pronk, PhD, Vivi Schlünssen, PhD, Zara A Stokholm, PhD, Henrik A Kolstad, PhD, Karin van Veldhoven, PhD, Ioannis Basinas, PhD, Martie van Tongeren,, Susan Peters, PhD

1. *Supplementary tables*
2. *Correspondence to: Karen Oude Hengel, PhD, TNO, Department of Work Health Technology, Postbus 3005, 2301 DA Leiden, The Netherlands. [E-mail: karen.oudehengel@tno.nl]*

Table S2.1 The proportion of job titles per risk category for all eight dimensions for Denmark, the Netherlands and the United Kingdom

	Denmark					The Netherlands					United Kingdom			
Dimensions	No risk	Low risk	Elevated risk	High risk		No risk	Low risk	Elevated risk	High risk		No risk	Low risk	Elevated risk	High risk
Number of people	15.9%	40.9%	32.7%	10.6%		22.0%	39.2%	18.3%	20.5%		14.6%	29.3%	33.5%	22.6%
Nature of contacts	16.1%	49.0%	26.8%	8.1%		22.0%	41.9%	30.5%	5.7%		15.0%	43.5%	35.8%	5.7%
Contaminated workspaces	16.1%	47.4%	7.5%	29.1%		23.2%	41.7%	12.6%	22.6%		28.9%	31.1%	9.3%	30.7%
Location	16.1%	4.3%	8.5%	71.1%		22.0%	11.0%	10.0%	57.1%		14.6%	9.3%	9.6%	66.5%
Social distancing	16.1%	36.0%	28.7%	19.3%		22.0%	39.0%	24.2%	14.8%		14.6%	42.9%	32.1%	10.4%
Face covering	17.7%	36.0%	39.6%	6.7%		22.0%	40.4%	36.6%	1.0%		14.6%	22.2%	61.4%	1.8%
Income insecurity	78.7%	10.8%	6.9%	3.7%		77.4%	12.0%	6.1%	4.5%		52.8%	43.9%	2.8%	0.4%
Migrant workers	43.7%	28.9%	15.7%	11.8%		4.9%	49.8%	36.6%	8.7%		0.8%	57.1%	40.9%	1.2%

Table S2.2 Agreements scores (95% CI), weighted kappa's (95% CI), total and variance within each country as result from step 1 (independent expert rating)

	Agreement score (95% CI)[1]	Weighted kappa (95% CI)	Total variance	Variance of total variance (%)	
				Job	Rater
Denmark					
Number	0.51 (0.48- 0.54)	0.55 (0.48- 0.63)	0.91	56.4	43.2
Nature of contacts	0.64 (0.62- 0.67)	0.60 (0.60- 0.60)	0.65	66.2	34.3
Contaminated workspaces	0.59 (0.57- 0.62)	0.66 (0.62- 0.69)	1.20	60.7	38.3
Location	0.70 (0.67- 0.72)	0.45 (0.30- 0.60)	1.31	45.8	55.0
Social distancing	0.55 (0.52- 0.58)	0.65 (0.62- 0.69)	0.90	65.4	34.6
Face covering	0.40 (0.37- 0.42)	-0.08 (-0.50- 0.34)	0.72	9.2	82.0
The Netherlands					
Number	0.53 (0.51- 0.56)	0.57 (0.53- 0.61)	1.21	57.9	43.6
Nature of contacts	0.68 (0.66- 0.71)	0.64 (0.62- 0.65)	0.79	64.8	35.3
Contaminated workspaces	0.57 (0.54- 0.59)	0.65 (0.61- 0.68)	1.23	63.6	36.3
Location	0.73 (0.71- 0.75)	0.65 (0.63- 0.67)	1.66	65.1	35.5
Social distancing	0.55 (0.52- 0.58)	0.64 (0.61- 0.68)	1.02	64.7	35.4
Face covering	0.39 (0.37- 0.42)	0.20 (0.06- 0.33)	0.68	23.9	71.4
United Kingdom					
Number	0.52 (0.50- 0.55)	0.47 (0.41- 0.53)	0.94	48.0	51.7
Nature of contacts	0.75 (0.73- 0.77)	0.67 (0.67- 0.67)	0.61	62.3	39.0
Contaminated workspaces	0.58 (0.55- 0.60)	0.62 (0.58- 0.65)	1.22	67.6	31.3
Location	0.74 (0.72- 0.76)	0.57 (0.52- 0.61)	1.37	57.1	43.7
Social distancing	0.62 (0.65- 0.46)	0.71 (0.71- 0.71)	0.72	71.0	28.7
Face covering	0.68 (0.66- 0.70)	0.43 (0.21- 0.65)	0.60	43.5	56.5

[1] 95%CI: 95% Confidence intervals.

Table S2.3. Agreements scores (95% CI), weighted kappa's (95% CI), total and variance within each country as result from step 2 (Group expert meeting and revision)

	Agreement score (95% CI)[1]	Weighted kappa (95% CI)	Total variance	Variance of total variance (%)	
				Job	Rater
Denmark					
Number	0.51 (0.49- 0.54)	0.50 (0.45- 0.56)	0.91	51.4	48.6
Nature of contacts	0.60 (0.57- 0.62)	0.59 (0.56- 0.62)	0.79	56.1	43.9
Contaminated workspaces	0.58 (0.56- 0.61)	0.55 (0.51- 0.60)	1.19	59.3	40.7
Location	0.68 (0.66- 0.70)	0.45 (0.38- 0.52)	1.54	46.0	54.0
Social distancing	0.47 (0.44- 0.49)	0.63 (0.59- 0.66)	1.07	62.7	37.3
Face covering	0.40 (0.38- 0.43)	0.37 (0.30- 0.45)	0.85	37.4	62.6
The Netherlands					
Number	0.52 (0.49- 0.54)	0.56 (0.52- 0.60)	1.24	57.3	42.7
Nature of contacts	0.69 (0.67: 0.72)	0.64 (0.64- 0.64)	0.77	64.8	35.2
Contaminated workspaces	0.57 (0.54- 0.59)	0.65 (0.61- 0.68)	1.23	64.5	35.5
Location	0.73 (0.71- 0.75)	0.65 (0.63- 0.67)	1.66	65.1	34.9
Social distancing	0.55 (0.52- 0.58)	0.64 (0.61- 0.68)	1.02	64.7	35.3
Face covering	0.56 (0.54- 0.59)	0.30 (0.19- 0.40)	0.73	33.2	66.8
United Kingdom					
Number	0.54 (0.52- 0.57)	0.55 (0.50- 0.60)	1.01	55.4	44.6
Nature of contacts	0.75 (0.73- 0.77)	0.67 (0.67- 0.67)	0.62	62.1	37.9
Contaminated workspaces	0.57 (0.55- 0.60)	0.61 (0.58- 0.65)	1.21	67.5	32.5
Location	0.74 (0.71- 0.76)	0.57 (0.52- 0.61)	1.37	57.1	42.9
Social distancing	0.62 (0.60- 0.65)	0.71 (0.71- 0.71)	0.72	71.1	28.9
Face covering	0.78 (0.76- 0.80)	0.54 (0.40- 0.69)	0.61	54.3	45.7

[1] 95%CI: 95% Confidence intervals.
